# Cyclosporin A Induces Cardiac Differentiation but Inhibits Hemato-Endothelial Differentiation of P19 Cells

**DOI:** 10.1371/journal.pone.0117410

**Published:** 2015-01-28

**Authors:** Seung-Cheol Choi, Hyunjoo Lee, Ji-Hyun Choi, Jong-Ho Kim, Chi-Yeon Park, Hyung-Joon Joo, Jae-Hyoung Park, Soon-Jun Hong, Cheol-Woong Yu, Do-Sun Lim

**Affiliations:** Department of Cardiology, Cardiovascular Center, Korea University Anam Hospital, Seoul, Korea; University of Tampere, FINLAND

## Abstract

Little is known about the mechanisms underlying the effects of Cyclosporin A (CsA) on the fate of stem cells, including cardiomyogenic differentiation. Therefore, we investigated the effects and the molecular mechanisms behind the actions of CsA on cell lineage determination of P19 cells. CsA induced cardiomyocyte-specific differentiation of P19 cells, with the highest efficiency at a concentration of 0.32 μM during embryoid body (EB) formation via activation of the Wnt signaling pathway molecules, Wnt3a, Wnt5a, and Wnt8a, and the cardiac mesoderm markers, Mixl1, Mesp1, and Mesp2. Interestingly, cotreatment of P19 cells with CsA plus dimethyl sulfoxide (DMSO) during EB formation significantly increases cardiac differentiation. In contrast, mRNA expression levels of hematopoietic and endothelial lineage markers, including Flk1 and Er71, were severely reduced in CsA-treated P19 cells. Furthermore, expression of Flk1 protein and the percentage of Flk1+ cells were severely reduced in 0.32 μM CsA-treated P19 cells compared to control cells. CsA significantly modulated mRNA expression levels of the cell cycle molecules, p53 and Cyclins D1, D2, and E2 in P19 cells during EB formation. Moreover, CsA significantly increased cell death and reduced cell number in P19 cells during EB formation. These results demonstrate that CsA induces cardiac differentiation but inhibits hemato-endothelial differentiation via activation of the Wnt signaling pathway, followed by modulation of cell lineage-determining genes in P19 cells during EB formation.

## Introduction

Cyclosporin A (CsA) is a powerful immunosuppressive drug that is widely used in organ transplantation and treatment of autoimmune disorders [1,2]. CsA suppresses T cell activity by forming a complex with the intracellular receptor, cyclophilin. This CsA-cyclophilin complex inhibits the calcium-dependent serine/threonine phosphatase, calcineurin, and subsequently blocks activity of nuclear factor of activated T cells (NFAT) [3–5]. The calcineurin/NFAT signaling pathway mediates multiple adaptive T-cell functions, and also contributes to innate immunity and regulates the homeostasis of innate cells [6].

Recently, CsA has been shown to have pleiotropic effects on stem cells, such as proliferation [7,8], survival [8], apoptosis [9,10], and differentiation [7,11,12]. Specifically, several findings on the effects of CsA resulting in enhanced cardiac differentiation have been reported. Sachinidis et al. [13] reported that 1 μM CsA increased the expression of Nkx2.5 and GATA4 in mouse embryonic stem (ES) cells, demonstrating that CsA has a procardiomyogenic effect. Yamashita and his colleagues [14] showed that 0.83–2.5 μM (1–3 μg/mL) of CsA induces cardiomyocyte differentiation of Flk1+ mesodermal cells but has no influence on the generation of Flk1+ mesoderm cells from undifferentiated ES cells; they demonstrated that among the progeny of Flk1+ mesoderm cells, CsA treatment is most effective in inducing the cardiac progenitors, FCV cells (Flk1+/CXCR4+/VE-cadherin- cell population) [15]. Similarly, they also demonstrated that 0.83–2.5 μM (1–3 μg/mL) of CsA enhances cardiac differentiation of Flk1+ mesodermal cells in mouse and human induced pluripotent stem (iPS) cells, with no effect on undifferentiated iPS cells [16]. Neither another calcineurin inhibitor, FK506, nor an NFAT inhibitor, 11R-VIVIT, reproduced the effect of CsA [14], indicating that the main cardiogenic effect of CsA may operate in an NFAT-independent fashion. Taken together, the effects of CsA on cardiomyogenic differentiation of pluripotent stem cells may be dependent on cell type, cell source, differentiation state, or culture condition, with beneficial effects occurring in a narrow window of CsA concentration. However, the molecular mechanisms behind the actions of CsA on the cardiomyogenic differentiation of stem cells are still unknown. Furthermore, little is known about the effects of CsA on the fate of stem cells, including cardiomyogenic differentiation.

The P19 embryonic stem cell line has been widely used as a model system for the study of molecular mechanisms underlying cardiomyogenic differentiation [17–20]. Cardiomyogenic differentiation of P19 cells has generally been induced by embryoid body (EB) formation in the presence of 0.5–1% dimethyl sulfoxide (DMSO) in non-adhesive Petri dishes [17]. To date, however, the efficacy of P19 cells to differentiate into a cardiomyogenic lineage remains low.

In this study, therefore, we used P19 cells to elucidate which signaling pathways are modulated during CsA-induced cell lineage specification, and to identify key lineage-determining genes that regulate CsA-induced cell lineage specification. We found that CsA induces specific cardiac differentiation of P19 cells via activation of the Wnt signaling pathway and cardiac mesoderm lineage markers, such as Mixl1, Mesp1, and Mesp2, at the expense of hemato-endothelial differentiation by inhibiting Flk1 and its related signaling molecules. Furthermore, cotreatment of P19 cells with CsA plus DMSO during EB formation significantly increases cardiac differentiation, and thus would be useful for the elucidation of the molecular mechanisms underlying the specification of stem cells to a cardiac lineage. Finally, we also demonstrated that CsA modulates cell cycle molecules and induces cell apoptosis during cell lineage specification of P19 cells.

## Materials and Methods

### Culture and cardiac differentiation of P19 cells

CsA (Sigma-Aldrich C3662, St. Louis, MO) was dissolved in ethanol at 10 mg/ml, and stored at -20°C until use. P19 cells were obtained from the American Type Culture Collection (ATCC, Rockville, MD) and cultured in Dulbecco’s Modified Eagle’s Medium (DMEM) (Invitrogen, Grand Island, NY) supplemented with 10% fetal bovine serum (FBS; Invitrogen). To induce differentiation, P19 cells were seeded at a density of 3 × 10^5^ cells in 10 ml of DMEM plus 20% FBS with different concentrations (0.08, 0.16, 0.32, 0.64, 1.25, 2.5, 5 or 10 μM) of CsA in the absence or presence of 1% DMSO (Sigma-Aldrich D4540) during EB formation in non-adhesive Petri dishes for 96 h. The formed EBs were transferred into 6-well culture plates, and cultured in DMEM with 20% FBS for a further 6–12 days. Medium was replaced every 2 days. Alternatively, different concentrations of CsA were added to the preformed EBs treated with or without 1% DMSO during EB formation. Images of beating cells were recorded on a Nikon inverted microscope equipped with a digital camera (Nikon, Tokyo, Japan). Niclosamide (Sigma-Aldrich N3150) was dissolved in DMSO at a 20 mM concentration and stored at -20°C. To inhibit Wnt signaling, P19 cells were pretreated with 0.2 μM niclosamide for 2 h and then treated with 0.32 μM CsA in the presence of 0.2 μM niclosamide for 48 h in DMEM plus 20% FBS during EB formation. After 48 h, P19 cells were further treated with 0.32 μM CsA in the absence of niclosamide for additional 48 h in DMEM plus 20% FBS. The formed EBs were transferred into 6-well culture plates, and cultured in DMEM with 20% FBS for an additional 6 days.

### Real-time PCR

Total RNA was extracted from cells using Trizol reagent (Invitrogen). The concentrations of total RNAs were determined using a Nanodrop 1000 spectrophotometer (Thermo Scientific, Waltham, MA). First-strand cDNA was synthesized from 0.5 μg of DNase-treated total RNA using 0.5 μg of random hexamers (Invitrogen), and 200 U of Moloney murine leukemia virus reverse transcriptase (Invitrogen) at 37°C for 60 min in a 20 μl volume. Real-time PCR was performed using a real-time PCR thermal cycler (Bio-Rad Laboratories, Hercules, CA). Each reaction contained 12.5 μl 2X SYBR Green PCR Mix (Bio-Rad Laboratories), 1.5 μl forward primer (5 μM), 1.5 μl reverse primer (5 μM), 5 μl of a 1:10 dilution of cDNA, and 4.5 μl H_2_O. To avoid genomic DNA amplification, intron-spanning primers were designed using ProbeFinder software (https://www.roche-applied-science.com). The primer sequences used for real-time PCR are listed in S1 Table. The measurement of gene expression was assayed in duplicate. Real-time PCR data were pooled from three independent experiments. Relative gene expression levels were quantified based on Ct and normalized to the reference gene, GAPDH.

### Immunocytochemistry

P19 cells were seeded at a density of 3 × 10^5^ cells per dish in 10 ml DMEM + 20% FBS in the presence of 0.32 μM CsA in non-adhesive Petri dishes for 96 h. The formed EBs were transferred onto 0.1% gelatin-coated coverslips in 12-well culture plates, and cultured in DMEM with 20% FBS for an additional 10 days. The cells were fixed in 4% paraformaldehyde for 10 min, permeabilized with 0.5% Triton X-100 for 30 min, washed in PBS + 0.1% Tween 20 (PBST), and preblocked with 5% normal goat serum (NGS; Invitrogen) in PBST for 1 h. The cells were stained with anti-alpha-myosin heavy chain (α-MHC; 1:400; Abcam ab15, Cambridge, MA), anti-cardiac troponin T (cTnT; 1:400; Thermo Scientific MS295), or anti-sarcomeric alpha-actinin (α-actinin; 1:400; Sigma-Aldrich A7811) antibody at 4°C overnight in 2% NGS in PBST, and then washed 3 times in PBST. To dissociate EBs into single cells, EBs collected at 48 or 96 h were washed with PBS and incubated with accutase (Sigma-Aldrich A6964) at 37°C for 5 min. After PBS washing, the cells were fixed in 4% paraformaldehyde for 10 min, washed in PBST, and preblocked with 5% NGS in PBST for 1 h. The cells were stained with anti-Flk1 (1:200; BD Pharmingen 555307, San Jose, CA) antibody at 4°C overnight in 2% NGS in PBST, and then washed 3 times in PBST. The cells were stained with Alexa Fluor 488-conjugated anti-rat IgG (1:1000; Molecular Probes, Eugene, Oregon) antibody for 1 h and washed 3 times in PBST. The nuclei were stained with DAPI (Sigma-Aldrich), and the cells were mounted using fluorescent mounting medium (Dako, Carpinteria, CA).

For dual-color immunostaining of EBs, the EBs were fixed in 4% paraformaldehyde for 15 min, and washed twice in PBST. The EBs were permeabilized with 0.5% Triton X-100 for 30 min, washed in PBST, and preblocked with 5% NGS in PBST for 2 h. The EBs were then stained with anti-Flk1 (1:200; BD Pharmingen 555307) antibody at 4°C overnight in 5% NGS in PBST and then washed three times for 30 min each with PBST. The EBs were then reblocked in 5% NGS in PBST for 2 h, and stained with Alexa Fluor 488 secondary antibody (1:1000; Molecular Probes) at 4°C overnight in 5% NGS in PBST and then washed three times for 30 min each with PBST. The EBs were stained with anti-Oct4 (1:100; Santa Cruz Biotechnology sc-5279, Santa Cruz, CA) antibody at 4°C overnight in 5% NGS in PBST and then washed three times for 30 min each with PBST. The EBs were reblocked in 5% NGS in PBST for 2 h, and stained with Alexa Fluor 594 secondary antibody (1:1000; Molecular Probes) plus 1 μg/ml of DAPI at 4°C overnight in 5% NGS in PBST and then washed three times for 30 min each with PBST. The EBs were mounted using fluorescent mounting medium. Fluorescence images were obtained using an inverted fluorescence microscope (Olympus, Tokyo, Japan) or were acquired with a confocal fluorescence microscope (Carl Zeiss LSM710, Oberkochen, Germany).

### Flow cytometry

EBs collected at 48 or 96 h were washed with PBS and incubated with accutase at 37°C for 5 min. After PBS washing, the cells were fixed with 2% paraformaldehyde for 10 min. The cells were then stained with anti-Flk1 (1:200; BD Pharmingen 555307) antibody diluted 1:100 in PBS containing 2% FBS, by incubating for 20 min at 4°C. For intracellular staining, the cells were permeabilized with ice-cold methanol for 20 min on ice and stained with anti-Oct4 (1:100; Santa Cruz Biotechnology sc-5279) antibody. The cells were incubated in PBS containing 2% FBS for 5 min and washed by centrifugation. The cells were then stained with a fluorescein isothiocyanate (FITC)-conjugated secondary (1:200; Sigma-Aldrich) antibody and incubated for 20 min at 4°C. After washing with PBS containing 2% FBS, the cells were resuspended and analyzed by flow cytometry. For negative control samples, all the conditions were kept the same, except that the primary antibody was omitted. Ten thousand cells per sample were analyzed on a flow cytometer (BD Biosciences, San Jose, CA). Dead cells and debris were gated out based upon the scattering properties of the cells. Data were analyzed using a software (BD Biosciences).

### Western blotting

EB formation was induced by plating 3 × 10^5^ cells in 10-cm non-adhesive Petri dishes in 10 ml DMEM supplemented with 20% FBS in the absence or presence of 0.32 μM of CsA. The cells were collected at 0, 12, 24, 48, 72, and 96 h after EB induction in the absence or presence of 0.32 μM of CsA. To examine the synergistic effect of CsA and DMSO on cardiac differentiation, EBs were formed by culturing P19 cells in suspension in non-adhesive Petri dishes for 4 days in the presence of 0.32 μM CsA, 1% DMSO, or 0.32 μM CsA + 1% DMSO, and EBs were then transferred to 6-well culture plates and cultured for an additional 12 days. P19 cells were rinsed with ice-cold PBS and lysed with ice-cold 1x cell lysis buffer (Cell Signaling Technology 9803, Danvers, MA) plus 1 mM phenylmethylsulfonyl fluoride. The protein concentrations of the samples were determined with a Bradford assay kit (Bio-Rad Laboratories). Ten μg each of the total protein samples was boiled in 2% SDS sample buffer for 5min and separated by electrophoresis on 10% SDS—polyacrylamide gel electrophoresis. After separation, the proteins were transferred to polyvinylidene fluoride membranes and blocked in TBST (10mM Tris, pH 8.0, 150mM NaCl and 0.1% Tween 20) with 5% non-fat dry milk for 1h. The membranes were rinsed and incubated with anti-α-MHC (1:1000; Abcam ab15), anti-p-Flk1 (1:1000; Cell Signaling Technology 2478), or anti-Flk1 (1:1000; Cell Signaling Technology 2479) antibody overnight at 4°C. Next, the membranes were washed three times for 20 min in TBST and incubated for 1 h with a horseradish peroxidase-conjugated secondary antibody (1:5000; Santa Cruz Biotechnology) in 3% non-fat dry milk. The reactive bands were visualized using ECL reagent (Thermo Scientific) and exposed to radiography film. The membranes were then stripped and reprobed with anti-GAPDH (1:10000 dilution; Sigma-Aldrich G8795) antibody as a loading control. Densitometric quantification of bands was carried out using a software (Bio-Rad Laboratories).

### Apoptosis assay and estimation of cell number

EBs collected at 48 or 96 h were washed with PBS, dissociated with accutase at 37°C for 5 min, and washed quickly with ice-cold PBS containing 2% FBS. Annexin V and propidium iodide (PI) staining were performed using the FITC Annexin V Apoptosis Detection Kit (BD Pharmingen 556570) according to the manufacturer’s instructions. To estimate the number of cells, P19 cells were seeded at 3 × 10^5^ cells in 10 ml DMEM + 20% FBS in the presence of 1% DMSO in non-adhesive Petri dishes for 48 or 96 h. The formed EBs were dissociated with accutase, washed with ice-cold PBS containing 2% FBS, and stained with 0.2% trypan blue (Invitrogen) to identify dead cells. Viable cells were then counted using a hemocytometer.

### Statistical analysis

All statistical values are expressed as the mean ± standard deviation (SD). Statistical analysis was performed using the Student’s t-test or Newman-Keuls test. Statistical significance was set at p < 0.05. All statistical analyses were performed using SigmaStat3.5 (SPSS, Chicago, IL).

## Results

### CsA induces cardiomyocyte-specific differentiation of P19 cells with the highest efficiency during EB formation

**Fig 1:**
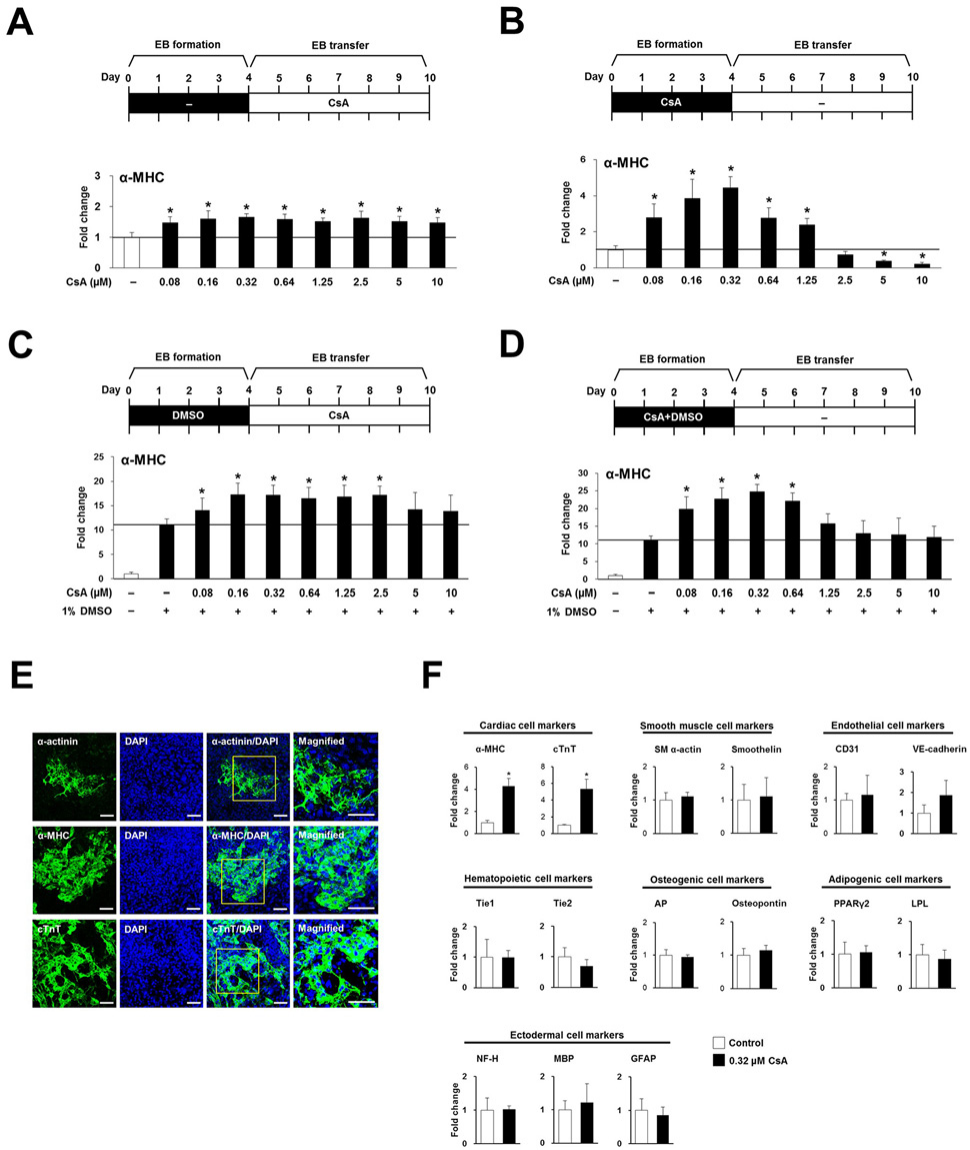
CsA induces cardiac differentiation of P19 cells during EB formation. Schematic diagrams for cardiac differentiation of P19 cells in the absence or presence of CsA or DMSO are shown in A, B, C and D. Real-time PCR was carried out with primers for the cardiac specific gene, α-MHC in A, B, C and D. All real-time PCR data represent mean ± SD from three independent experiments (*p<0.05). (A) Effect of CsA on cardiomyocyte differentiation of mesodermal lineage cells. EBs were formed by culturing P19 cells in suspension in non-adhesive Petri dishes for 4 days; the EBs were transferred to 6-well culture plates and cultured for an additional 6 days with different concentrations of CsA. (B) Effect of CsA on mesodermal lineage differentiation of undifferentiated P19 cells. EBs were formed by culturing P19 cells in suspension in non-adhesive Petri dishes for 4 days with different concentrations of CsA, and EBs were transferred to 6-well culture plates and cultured for an additional 6 days. (C) Effect of CsA on cardiomyocyte differentiation of DMSO-induced mesodermal lineage cells. EBs were formed by culturing P19 cells in suspension in non-adhesive Petri dishes for 4 days with 1% DMSO, and EBs were transferred to 6-well culture plates and cultured for an additional 6 days with different concentrations of CsA. (D) Synergistic effect of CsA and DMSO on mesodermal lineage differentiation of undifferentiated P19 cells. EBs were formed by culturing P19 cells in suspension in non-adhesive Petri dishes for 4 days either with or without different concentrations of CsA and 1% DMSO, and EBs were transferred to 6-well culture plates and cultured for an additional 6 days. (E) Immunofluorescence images showing cardiac differentiation of P19 cells by 0.32 μM CsA treatment during EB formation. EBs formed by culturing P19 cells in suspension in non-adhesive Petri dishes for 4 days with 0.32 μM CsA were further cultured for 6 days in 6-well culture plates. Expression of the cardiomyocyte specific markers, sarcomeric α-actinin, cTnT, and α-MHC was assessed by immunostaining, and nuclei were stained with DAPI. Scale bars = 50 μm. (F) Real-time PCR showing cardiac specific differentiation of P19 cells in response to 0.32 μM CsA treatment during EB formation. EBs formed by culturing P19 cells in suspension in non-adhesive Petri dishes for 4 days without (control) or with 0.32 μM CsA were further cultured for 6 days in 6-well culture plates. α-MHC, α-alpha-myosin heavy chain; AP, alkaline phosphatase; cTnT, cardiac troponin T; EB, embryoid body; GFAP, glial fibrillary acidic protein; LPL, lipoprotein lipase; MBP, myelin basic protein; NF-H, neurofilament-H; PPARγ, peroxisome proliferator-activated receptor γ; SM α-actin, smooth muscle alpha-actin.

A previous study reported that CsA induces the expression of cardiac markers and generated beating aggregates during EB formation [21]. However, a contrasting study reported that CsA induces cardiomyocyte differentiation from Flk1+ mesodermal cells but has no influence on Flk1+ mesoderm cell generation from undifferentiated ES cells [14]. Therefore, we wanted to determine whether CsA plays a role in specification to a mesodermal cell lineage from undifferentiated stem cells or on cardiomyocyte differentiation from mesodermal lineage cells. To do this, we investigated the effects of CsA on cardiac differentiation of P19 cells under different conditions. First, we examined the effects of CsA on cardiomyocyte differentiation of preformed EBs. EB formation was induced in the absence of CsA, and the formed EBs were then transferred into 6-well culture plates, and cultured in DMEM + 20% FBS for an additional 6 days with 0, 0.08, 0.16, 0.32, 0.64, 1.25, 2.5, 5, or 10 μM CsA (Fig. 1A). CsA addition after EB formation moderately but significantly increased the expression of α-MHC mRNA, a cardiac cell-specific molecule, at the examined CsA concentrations (Fig. 1A).

Second, we investigated whether CsA triggers specification of undifferentiated P19 cells into a cardiac cell lineage. EB formation was induced in the presence of 0, 0.08, 0.16, 0.32, 0.64, 1.25, 2.5, 5, or 10 μM CsA during EB formation, and then the formed EBs were transferred into 6-well culture plates and cultured in DMEM + 20% FBS for an additional 6 days (Fig. 1B). At concentrations from 0.08 to 1.25 μM, CsA significantly induced cardiac differentiation of P19 cells, with the highest expression of α-MHC mRNA found in 0.32 μM CsA-treated P19 cells (Fig. 1B). However, conversely, high concentrations (5 and 10 μM) of CsA decreased cardiac differentiation of P19 cells (Fig. 1B). This result shows that CsA induces cardiac differentiation of P19 cells, and plays an important role in inducing cardiac differentiation of P19 cells at the EB formation stage rather than at the post-EB formation stage.

Third, we examined whether CsA has a synergistic effect on cardiomyocyte differentiation of DMSO-treated EBs. P19 cell aggregates were formed in non-adhesive Petri dishes for 96 h in the presence of 1% DMSO, and the formed EBs were then transferred into 6-well culture plates, and cultured in DMEM + 20% FBS for an additional 6 days in the presence of 0, 0.08, 0.16, 0.32, 0.64, 1.25, 2.5, 5, or 10 μM CsA (Fig. 1C). DMSO treatment during EB formation and consecutive CsA administration to the preformed EBs moderately but significantly increased cardiac differentiation of P19 cells treated with 0.08 to 2.5 μM CsA relative to the DMSO only-treated group. This result shows that DMSO treatment during EB formation, followed by administration of CsA to the preformed EBs, moderately increases the cardiac differentiation of P19 cells.

Fourth, we determined whether the combined treatment of CsA plus DMSO has a synergistic effect during EB formation. EB formation was induced in the presence of 0, 0.08, 0.16, 0.32, 0.64, 1.25, 2.5, 5 or 10 μM CsA and 1% DMSO during EB formation, and the formed EBs were then transferred into 6-well culture plates and cultured in DMEM + 20% FBS for an additional 6 days (Fig. 1D). Combined treatment of P19 cells with both DMSO and 0.08, 0.16, 0.32, or 0.64 μM CsA during EB formation significantly increased expression of α-MHC mRNA compared with the DMSO only-treated group, showing the synergistic effects of CsA in combination with DMSO (Fig. 1D). However, the combined administration of more than 5 μM of CsA plus DMSO during EB formation had no effect on cardiac differentiation of P19 cells. Taken together, these results show that CsA induces cardiac differentiation of P19 cells at the EB formation stage as well as at the post-EB formation stage.

We used 0.32 μM CsA for subsequent experiments because we found it to be the optimal concentration for differentiation into a cardiac cell lineage during EB formation. To further confirm cardiomyogenic differentiation in 0.32 μM CsA-treated P19 cells, immunostaining with antibodies against cardiac-specific markers, namely sarcomeric α-actinin, α-MHC, and cTnT was performed. A number of cells showing specific staining patterns for cardiac-specific markers were observed among P19 cells treated with 0.32 μM CsA during EB formation (Fig. 1E). Moreover, we carried out real-time PCR analysis with different cell lineage markers to investigate whether P19 cells are specifically differentiated into a cardiac lineage by 0.32 μM CsA treatment during EB formation. We confirmed that 0.32 μM CsA-treated P19 cells are specifically differentiated into cardiac cell lineage. However, no significant degree of differentiation into smooth muscle, endothelial, hematopoietic, osteogenic, adipogenic, or ectodermal cell lineages was detected (Fig. 1F).

The sizes of EBs treated with 10 μM CsA (S1A Fig.) or 10 μM CsA plus 1% DMSO (S1B Fig.) during EB formation were smaller than those of EBs formed in other concentrations of CsA or of the control group, suggesting that 10 μM CsA may inhibit EB formation. Furthermore, some EBs formed from 10 μM CsA-treated P19 cells were attached to the dish surfaces or showed a distorted rod-like morphology (S1C Fig.), although we used non-adhesive Petri dishes to avoid adhesion of EBs to the plastic surfaces. However, EB attachment was not observed in the group treated with 10 μM CsA plus 1% DMSO, indicating that EB adhesiveness caused by 10 μM CsA was abolished by 1% DMSO.

### CsA induces cardiac differentiation via activation of the Wnt/β-catenin signaling during EB formation

**Fig 2:**
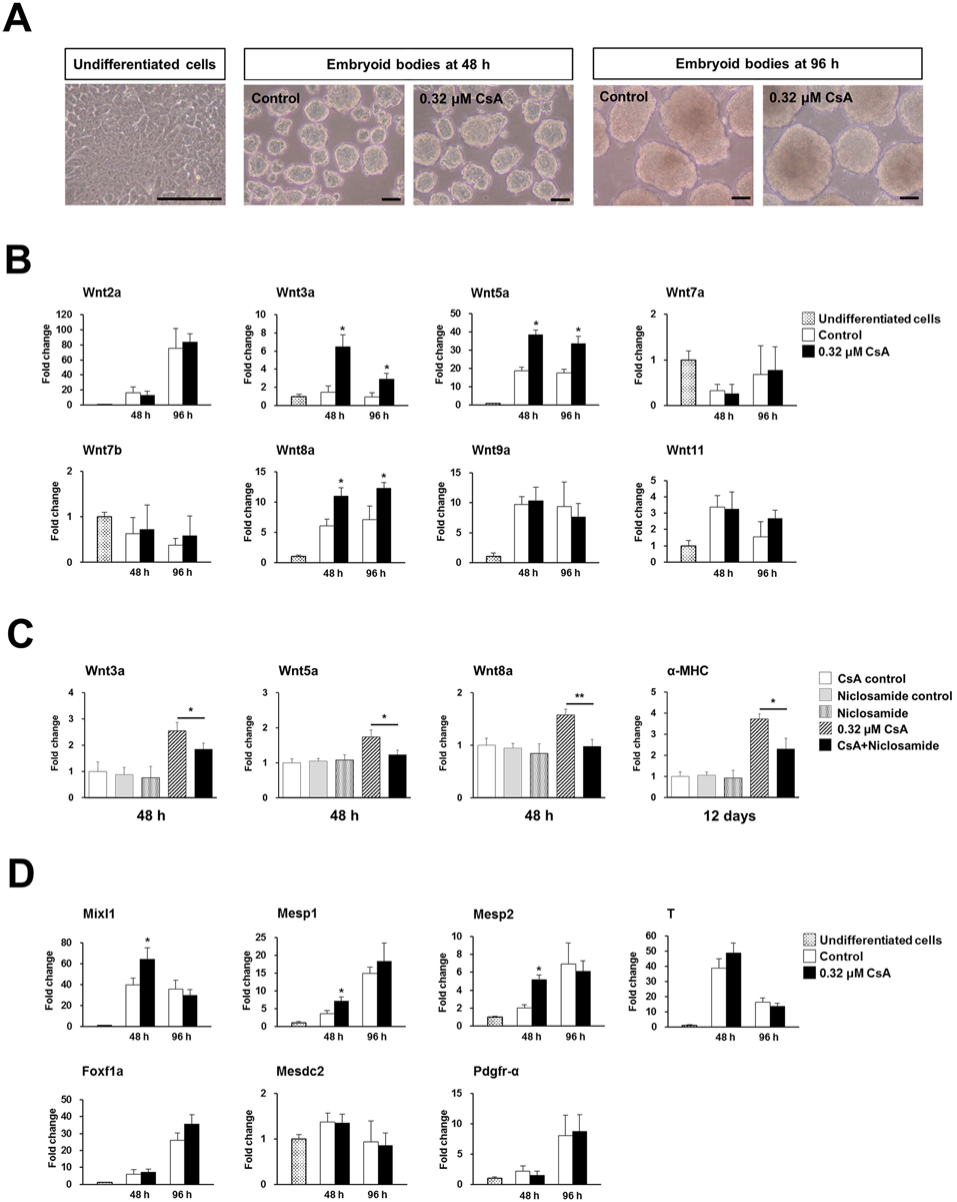
CsA induces cardiac differentiation via activation of the Wnt/β-catenin signaling during EB formation. EBs were formed by culturing P19 cells in suspension in non-adhesive Petri dishes for 48 or 96 h in the absence (control) or presence of 0.32 μM CsA. (A) Morphology of undifferentiated P19 cells and EBs treated with 0.32 μM CsA for 48 or 96 h. Scale bars = 100 μm. (B) Real-time PCR result showing upregulation of Wnt3a, Wnt5a, and Wnt8a mRNAs in 0.32 μM CsA-treated P19 cells during EB formation. Data represent mean ± SD from three independent experiments (*p<0.05). (C) Real-time PCR results showing significant reduction of 0.32 μM CsA-induced Wnt3a, Wnt5a, Wnt8a and of α-MHC mRNAs by niclosamide, a potent inhibitor of Wnt signaling. Following pretreatment with 0.2 μM niclosamide for 2 h, P19 cells were treated with 0.32 μM CsA in the presence of 0.2 μM niclosamide for 48 h in DMEM plus 20% FBS during EB formation. After 48 h, P19 cells were further treated with 0.32 μM CsA in the absence of niclosamide for an additional 48 h during EB formation. The formed EBs were transferred into 6-well culture plates, and cultured in DMEM with 20% FBS for a further 6 days. CsA control indicates time-matched vehicle control (0.004% ethanol). Niclosamide control indicates time-matched vehicle control (0.0005% DMSO). Data represent mean ± SD from three independent experiments (*p<0.05; **p<0.01). (D) Real-time PCR results showing upregulation of the cardiac mesoderm regulator genes, Mixl1, Mesp1, and Mesp2 in 0.32 μM CsA-treated P19 cells during EB formation. Data represent mean ± SD from three independent experiments (*p<0.05).

Noncanonical and canonical Wnt/β-catenin signaling is known to be required to enhance cardiac differentiation [22–25]. Therefore, we investigated the possible role of the Wnt/β-catenin signaling pathway in 0.32 μM CsA-treated P19 cells during EB formation by examining the mRNA levels of different Wnt molecules by real-time PCR. No distinct morphological change was observed EBs treated with 0.32 μM CsA for 48 or 96 h (Fig. 2A). Wnt3a, Wnt5a, and Wnt8a mRNA levels were significantly induced at 48 and 96 h by 0.32 μM CsA treatment during EB formation by P19 cells (Fig. 2B). However, significant differences were not found in Wnt2a, Wnt7a, Wnt7b, Wnt9a, or Wnt11 mRNA expression in the 0.32 μM CsA-treated group, compared to the control (Fig. 2B). Niclosamide, a potent inhibitor of the Wnt/β-catenin signaling [26] was used to prove whether Wnt/β-catenin signaling was involved in CsA-induced cardiac differentiation. The mRNA levels of Wnt3a, Wnt5a, and Wnt8a induced by 0.32 μM CsA during EB formation of P19 cells were significantly reduced by cotreatment with 0.2 μM niclosamide (Fig. 2C). Furthermore, cotreatment of niclosamide with CsA during EB formation significantly reduced the mRNA level of α-MHC, a cardiomyocyte marker, at 12 days of differentiation, indicating that activation of Wnt signaling pathway is involved in CsA-induced cardiac differentiation of P19 cells (Fig. 2C).

The BMP/Smad signaling pathway is known to be essential for the cardiac differentiation of ES cells, including P19 cells [18,27]. Therefore, quantitative analysis of mRNA expression of BMP/Smad pathway molecules in 0.32 μM CsA-treated P19 cells was carried out to investigate which molecules, if any, were modulated by CsA treatment. However, we did not find mRNA expression levels of BMP/Smad signaling pathway molecules in 0.32 μM CsA-treated P19 cells to be significantly affected (S2A Fig.). In addition to the BMP/Smad signaling pathway, we also examined mRNA levels of TGF-β superfamily signaling pathway molecules, namely TGF-β, Nodal, Gdf, and their receptors, in P19 cells treated with 0.32 μM CsA during EB formation, because TGF-β superfamily signaling pathways are well known to control cellular differentiation [28]. Among the TGF-β, Nodal, and GDF1 signaling pathway molecules examined in this study, only the mRNA level of Tgfb1 was increased, at a modest but significant level at 48 h in P19 cells treated with 0.32 μM CsA during EB formation (S2B Fig.). However, Nodal and GDF1 signaling pathway molecules were not affected overall in P19 cells treated with 0.32 μM CsA during EB formation (S2B Fig.).

We investigated the effects of CsA on mesendoderm specification during EB formation of P19 cells by quantitative real-time PCR. The cardiac mesoderm markers Mesp1, Mesp2, and Mixl1 were upregulated at modest but significant levels at 48 h in P19 cells treated with 0.32 μM CsA during EB formation (Fig. 2D). However, CsA treatment did not significantly affect mRNA expression levels of other investigated mesodermal lineage markers, namely T, Foxf2a, Mesdc2, and Pdgfr-α during cell lineage specification of P19 cells (Fig. 2D). The mRNA expression levels of the endodermal lineage markers Cdh1, Cxcr4, Dab2, Eomes, Foxh1, Gata6, and GCNF were not significantly modulated by CsA treatment during EB formation by P19 cells (S3 Fig.).

These results show that CsA induces specification into a cardiac mesodermal lineage via activation of the Wnt/β-catenin signaling pathway.

### Cotreatment of P19 cells with CsA plus DMSO during EB formation synergistically increases cardiac differentiation

**Fig 3:**
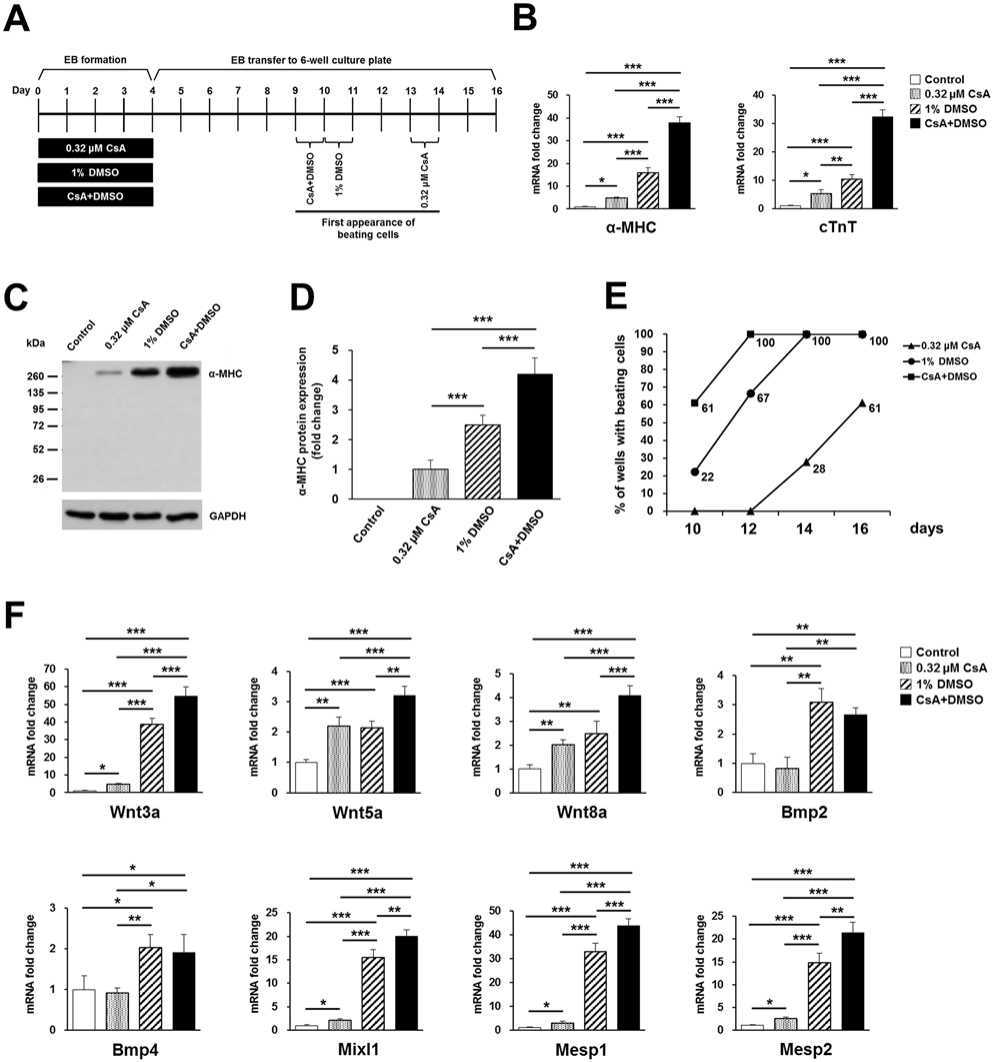
CsA and DMSO synergistically increase cardiac differentiation of P19 cells during EB formation. (A) Schematics for cardiac differentiation of P19 cells treated with 0.32 μM CsA, 1% DMSO, or CsA plus DMSO are shown. (B) Real-time PCR results showing the synergistic effect on cardiac differentiation of P19 cells at day 16 of differentiation after cotreatment with 0.32 μM CsA and 1% DMSO during EB formation. Data represent mean ± SD from three independent experiments (*p<0.05; **p<0.01; ***p<0.001). (C) Representative Western blot showing the synergistic effect on cardiac differentiation of P19 cells of cotreatment with 0.32 μM CsA and 1% DMSO. The cell lysates were probed with anti-α-MHC antibody, and with anti-GAPDH antibody as a loading control. (D) The relative expression levels of α-MHC protein were quantified by densitometric analysis and normalized against the protein expression levels of GAPDH, used as a loading control. The results shown represent mean ± SD from three independent experiments (***p<0.001). (E) Graph showing the percentages of 6-well culture plates containing beating cells with 0.32 μM CsA, 1% DMSO, or CsA plus DMSO. (F) Real-time PCR results showing the synergistic effect of Wnt3a, Wnt5a, Wnt8a, Mixl1, Mesp1 and Mesp2 mRNA expression in EBs treated for 48 h with 0.32 μM CsA and 1% DMSO. Data represent mean ± SD from three independent experiments (*p<0.05; **p<0.01; ***p<0.001).

In this study, we found a significant increase in mRNA expression levels of α-MHC following combined administration of 0.08, 0.16, 0.32, or 0.64 μM CsA and 1% DMSO during EB formation by P19 cells (Fig. 1D). Therefore, we further investigated the synergistic effect of 0.32 μM CsA plus 1% DMSO on cardiac differentiation by real-time PCR, Western blotting, and assaying beating activity. Schematics for cardiac differentiation of P19 cells treated with 0.32 μM CsA, 1% DMSO, or CsA plus DMSO during EB formation are shown (Fig. 3A). The mRNA expression levels of a cardiomyocyte marker, α-MHC, were significantly increased at day 16 of differentiation in cells treated during EB formation with 0.32 μM CsA- (4.8-fold), 1% DMSO- (15.9-fold), or CsA plus DMSO-treated group (37.8-fold), compared to the untreated control group (Fig. 3B). Similarly, the mRNA expression levels of another cardiomyocyte marker, cTnT were upregulated at day 16 of differentiation in cells treated during EB formation with 0.32 μM CsA (5.2-fold), 1% DMSO (10.5-fold), or CsA plus DMSO (32.4-fold), compared to the untreated control group (Fig. 3B). Furthermore, we detected α-MHC protein in the 0.32 μM CsA, 1% DMSO, and CsA plus DMSO-treated groups but not in the untreated control at day 16 of differentiation (Fig. 3C). Expression levels of α-MHC protein were significantly increased in the 1% DMSO- (2.5-fold), and CsA plus DMSO-treated groups (4.2-fold) relative to the 0.32 μM CsA-treated group (Fig. 3D). After confirming the upregulated expression of cardiomyocyte-specific markers in cells receiving combined treatment with CsA plus DMSO at both the mRNA and protein level, we then examined the incidence of beating cells. Beating cells were first observed approximately at day 9–10, 10–11, and 13–14 of differentiation in CsA plus DMSO, 1% DMSO and 0.32 μM CsA, respectively (Fig. 3A). Moreover, 100% (18 of 18) of the wells contained beating cells at day 12 of differentiation in CsA plus DMSO, and at day 14 of differentiation in DMSO only (Fig. 3E). About 61% (11 of 18) of wells contained beating cells in the 0.32 μM CsA-treated group (Fig. 3E), but no beating cells were found in control wells. In addition, small beating cell clusters were mainly found among the 0.32 μM CsA-treated P19 cells (S1 Video), whereas large beating cell clusters were observed in the CsA plus DMSO-treated group (S2 Video), compared to the CsA- (S1 Video) or DMSO-treated (S3 Video) group.

Next, we wanted to elucidate the molecules involved in enhancement of cardiac differentiation induced by combined treatment with CsA plus DMSO. Therefore, we investigated whether the Wnt molecules and cardiac mesodermal markers upregulated by CsA treatment during EB formation are further activated by cotreatment with CsA plus DMSO. At 48 h post-EB formation, the expression of the Wnt molecules, Wnt3a, Wnt5a, and Wnt8a were more upregulated by combined treatment with CsA plus DMSO compared to CsA only- or DMSO only-treated cells (Fig. 3F). Similarly, the mRNA expression levels of the cardiac mesodermal markers, Mixl1, Mesp1, and Mesp2 were significantly increased in CsA plus DMSO-treated cells compared to CsA only- or DMSO only-treated cells (Fig. 3F). However, mRNA expression levels of Bmp2 and Bmp4 were not further activated by combined treatment with CsA plus DMSO compared to DMSO alone although they were significantly upregulated at 48 h in 1% DMSO-treated P19 cells (Fig. 3F). Interestingly, EBs composed of more tightly packed cells were observed in the 1% DMSO- and CsA plus DMSO-treated groups compared to the untreated or 0.32 μM CsA-treated groups (S4 Fig.).

Taken together, these results showed that CsA and DMSO synergistically increased cardiac differentiation, possibly via activation of the Wnt signaling pathway molecules and cardiac mesoderm markers.

### CsA severely inhibits differentiation into hemato-endothelial cell lineages by blocking Er71- and Flk1-dependent pathways during EB formation

**Fig 4:**
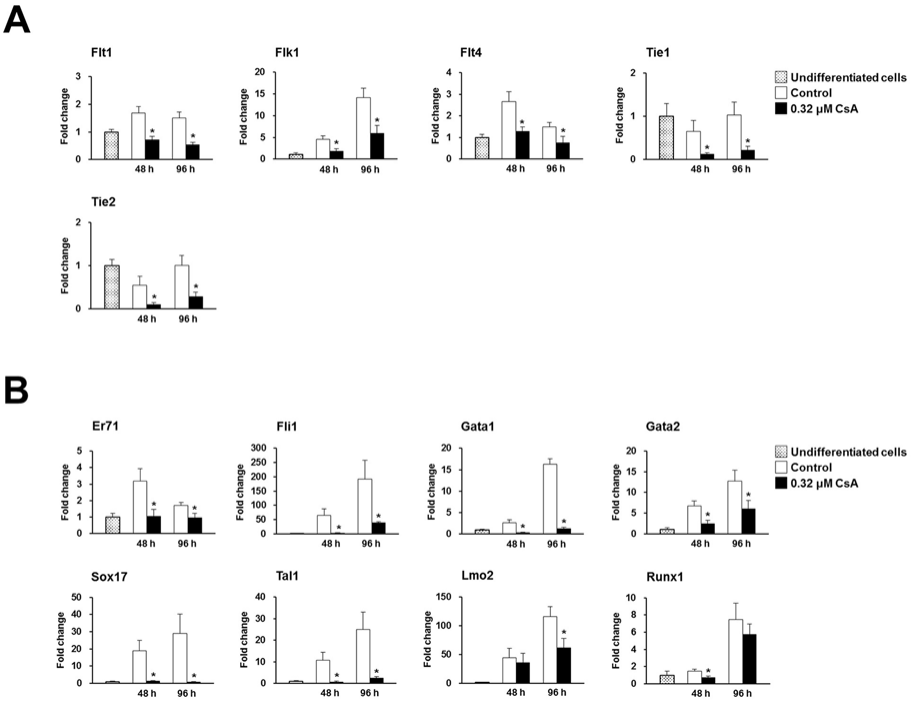
CsA severely inhibits expression of endothelial and hematopoietic lineage markers during EB formation. Real-time PCR result showing significant inhibition into endothelial (A) and hematopoietic (B) lineage markers in P19 cells treated with 0.32 μM CsA during EB formation. Data represent mean ± SD from three independent experiments (*p<0.05).

We also investigated the effects of CsA on endothelial and hematopoietic cell lineage specification during EB formation by P19 cells via quantitative real-time PCR. Interestingly, the mRNA expression levels of endothelial lineage markers, namely VEGF receptors (Flk1, Flt1 and Flt4), Tie1, and Tie2 were dramatically reduced at both 48 and 96 h in 0.32 μM CsA-treated P19 cells (Fig. 4A). Moreover, the mRNA expression levels of the hematopoietic lineage markers Er71, Fli1, Gata1, Gata2, Sox17, Tal1, Lmo2, and Runx1 were also severely reduced in P19 cells treated with 0.32 μM CsA during EB formation (Fig. 4B).

Flk1, the gene encoding vascular endothelial growth factor receptor 2 (VEGFR-2), is known to be a key player in normal hematopoiesis and vasculogenesis [29]. In light of this fact, we further investigated the modulation of the Flk1 gene at the protein level during the differentiation time course, because severe reduction of Flk1 mRNA was observed in P19 cells treated with 0.32 μM CsA during EB formation.

**Fig 5:**
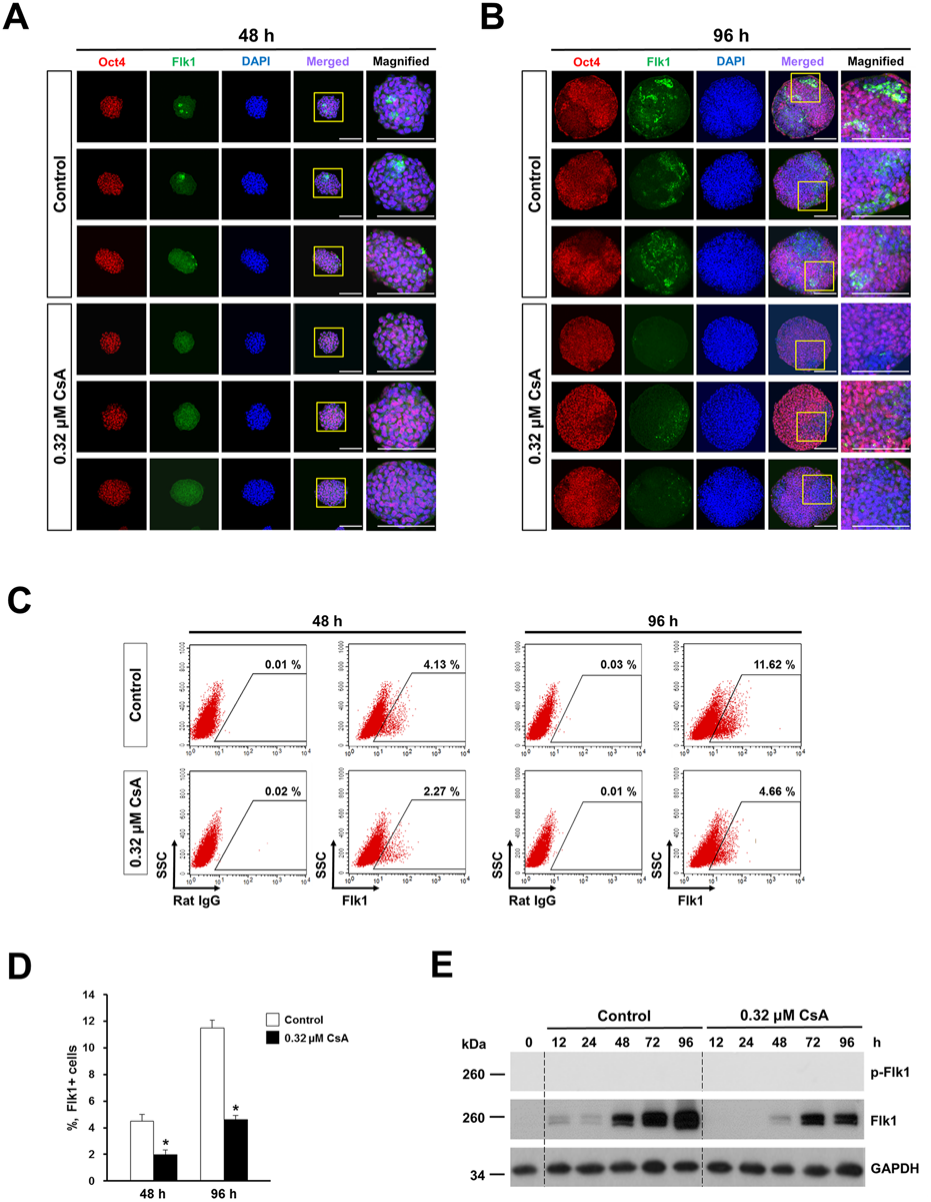
CsA reduces Flk1 protein expression and significantly decreases the percentages of Flk1+ cells. Immunofluorescence staining showing cells with strong expression of Flk1 and weak expression of Oct4 in EBs treated with 0.32 μM CsA for 48 (A) and for 96 h (B). Boxed regions are magnified in the right panels. Scale bars = 100 μm. (C) Representative flow cytometry profiles showing the decreased percentages of Flk1+ cells in P19 cells treated with 0.32 μM CsA during EB formation for 48 and 96 h, respectively. (D) Flow cytometric quantification of Flk1+ cells at 48 and 96 h after 0.32 μM CsA treatment during EB formation by P19 cells. Data represent mean ± SD from three independent experiments (*p<0.05). (E) Western blot showing strong reduction of Flk1 protein expression in P19 cells treated with 0.32 μM CsA during EB formation.

We found that Flk1 expression was dramatically reduced in 0.32 μM CsA-treated EBs at 48 and 96 h compared to the untreated control as determined by immunostaining (Fig.s 5A and 5B; S5 Fig.). The intensity of Flk1 expression was inversely correlated with that of Oct4 expression in 0.32 μM CsA-treated EBs as well as in untreated EBs at 48 and 96 h (Fig.s 5A and 5B). Furthermore, the percentages of Flk1+ cells were significantly lower at both 48 and 96 h with 0.32 μM CsA treatment during EB formation by P19 cells (Fig.s 5C and 5D). Flk1 protein was weakly expressed at 12 h and upregulated from 48 to 96 h in untreated P19 cells (Fig. 5E). However, expression of Flk1 protein was completely blocked from 12 to 24 h in 0.32 μM CsA-treated P19 cells. Flk1 protein was first detected at 48 h, and reduced expression levels of Flk1 protein were sustained from 72 to 96 h in 0.32 μM CsA-treated P19 cells compared to untreated P19 cells (Fig. 5E). Phosphorylated Flk1 was not detected in untreated control and 0.32 μM CsA-treated P19 cells (Fig. 5E).

These results show that CsA severely inhibits differentiation into endothelial and hematopoietic cell lineage possibly via blocking Er71- and Flk1-dependent pathways during EB formation in P19 cells.

### CsA differentially modulates expression of stem cell markers in a differentiation time-specific manner

**Fig 6:**
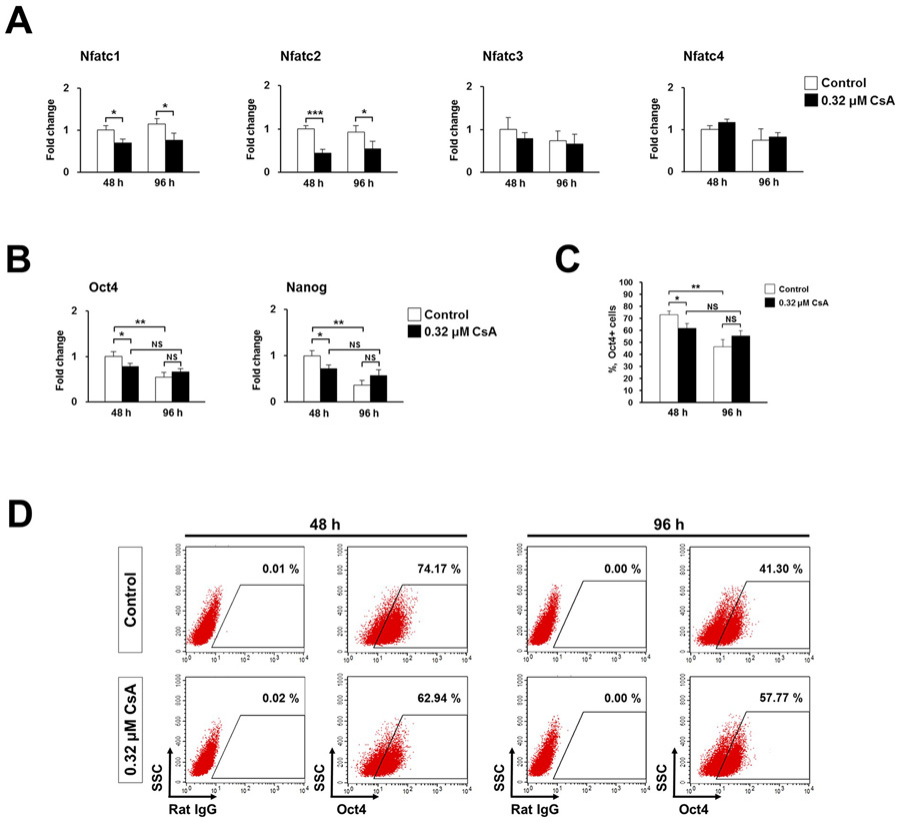
CsA differently modulates expression of stem cell markers in a differentiation stage-specific manner possibly via inhibition of the calcineurin/NFAT signaling pathway. (A) Real-time PCR result showing reduced expression of NFATc transcription factors in P19 cells treated with 0.32 μM CsA during EB formation. Data represent mean ± SD from three independent experiments (*p<0.05; ***p<0.001). (B) Real-time PCR result showing modulated expression of stem cell markers in P19 cells treated with 0.32 μM CsA during EB formation. Data represent mean ± SD from three independent experiments (*p<0.05; **p<0.01). NS, not significant. (C) The percentages of Oct4+ cells determined by flow cytometry at 48 and 96 h after 0.32 μM CsA treatment during EB formation by P19 cells. Data represent mean ± SD from three independent experiments (*p<0.05; **p<0.01). NS, not significant. (D) Representative flow cytometry plots showing different expression patterns of Oct4+ cells at 48 and 96 h after 0.32 μM CsA treatment during EB formation by P19 cells.

It is well known that CsA inhibits calcineurin activity and subsequently NFATc activation, resulting in immunosuppression by blocking the growth and differentiation of T cells [3–6]. Li et al. [30] reported that the calcineurin/NFAT signaling critically regulates early lineage specification in mouse ES cells and embryos. Moreover, CsA can maintain the expression of Oct4 and Nanog as well as sustain a growth rate of ES cells by inhibiting the calcineurin/NFAT pathway [30]. In this context, we used quantitative real-time PCR to determine whether 0.32 μM CsA treatment during EB formation by P19 cells affects mRNA expression levels of NFATc transcription factors. Out of four NFATc members, the expression levels of Nfatc1 and Nfatc2 mRNA were significantly downregulated at both 48 and 96 h in 0.32 μM CsA-treated P19 cells (Fig. 6A). However, expression of Nfatc3 and Nfatc4 mRNA was not affected by CsA treatment during EB formation by P19 cells (Fig. 6A).

Next, we examined the mRNA expression levels of stem cell markers after CsA treatment, based on previous reports that CsA sustains stemness of ES cells via regulation of the calcineurin/NFAT signaling pathway [30]. Furthermore, it is well known that downregulated expression of stem cell markers is an indicator of differentiation. The mRNA expression levels of the stem cell markers, Oct4 and Nanog, were significantly downregulated at 48 h in 0.32 μM CsA-treated P19 cells compared to the untreated control. Although their expressions increased, conversely, at 96 h in 0.32 μM CsA-treated P19 cells, this difference did not reach statistical significance (Fig. 6B). Furthermore, the percentages of Oct4+ cells were significantly decreased at 48 h but conversely increased at 96 h in 0.32 μM CsA-treated P19 cells (Fig. 6C and 6D). Interestingly, Oct4 and Nanog mRNA levels showed no significant differences between 48 and 96 h in 0.32 μM CsA-treated P19 cells, whereas they were significantly reduced at 96 h compared to 48 h in the untreated controls (Fig. 6B). Moreover, there were no significant differences in the percentages of Oct4+ cells between 48 and 96 h among 0.32 μM CsA-treated P19 cells, but the percentage of Oct4+ cells was significantly reduced at 96 h compared to 48 h, in the untreated control (Fig. 6C and 6D).

These results show that CsA treatment significantly downregulates expression of stem cell markers at 48 h, but their expressions were conversely increased at 96 h during EB formation indicating that CsA may modulate expression of stem cell markers via inhibition of the calcineurin/NFAT signaling pathway in a differentiation stage-specific manner.

### CsA modulates cell cycle molecules and induces cell death during EB formation by P19 cells

**Fig 7:**
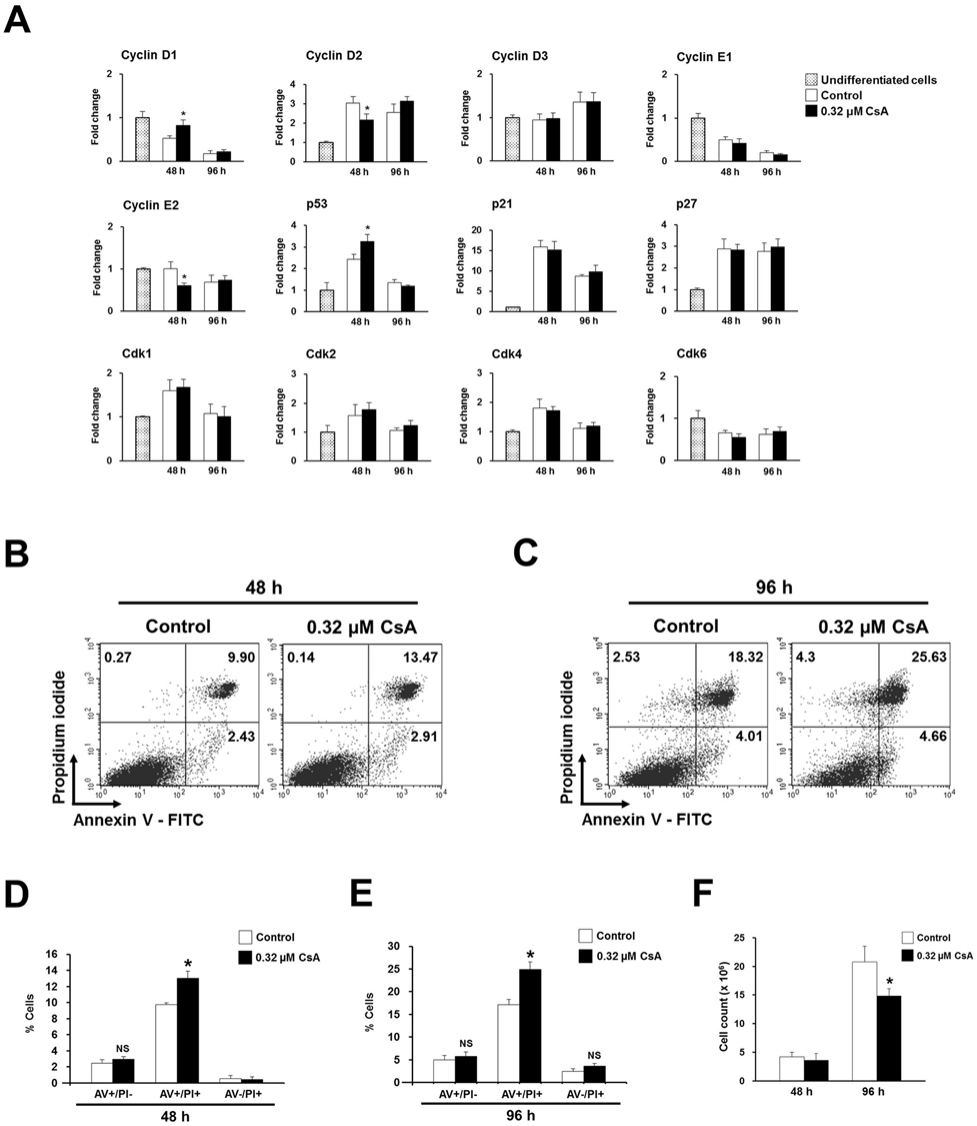
Treatment of P19 cells with CsA during EB formation modulates mRNA expression levels of cell cycle molecules and reduces cell number. (A) Real-time PCR result showing significant modulation of mRNA expression of Cyclins D1, D2, and E2, and p53 in P19 cells treated with 0.32 μM CsA during EB formation. (B) Representative flow cytometry plots showing annexin V+/PI+ apoptotic cells at 48 (B) and 96 h (C) in P19 cells treated with 0.32 μM CsA during EB formation. Bar graph of three independent experiments showing the percentages of annexin V+/PI+ apoptotic cells in P19 cells treated with 0.32 μM CsA during EB formation for 48 (D) and 96 h (E). (F) Estimation of the number of viable cells at 48 and 96 h in P19 cells treated with 0.32 μM CsA during EB formation.

It is well known that components of the cell cycle machinery play an important role during stem cell differentiation [20,31–35]. Therefore, we investigated which cell cycle molecules are involved in CsA-induced cardiac differentiation of P19 cells during EB formation by quantitative real-time PCR. CsA significantly modulated the mRNA expression levels of Cyclins D1, D2 and E2 at 48 h (Fig. 7A). Interestingly, the mRNA level of p53 was also significantly upregulated at 48 h in 0.32 μM CsA-treated EBs (Fig. 7A). However, the Cdks, Cdk1, Cdk2, Cdk4, and Cdk6 were not affected by CsA treatment during EB formation (Fig. 7A).

We also examined the effect of CsA on apoptosis during EB formation by P19 cells via flow cytometry-based annexin V/PI staining. The percentages of annexin V+/PI+ cells representing late apoptotic and necrotic cells were significantly increased in 0.32 μM CsA-treated P19 cells at both 48 (Fig. 7B and 7D) and 96 h (Fig. 7C and 7E). However, the percentages of annexin V+/PI- cells representing early apoptotic cells slightly increased at non-significant levels in 0.32 μM CsA-treated P19 cells at both 48 (Fig. 7B and 7D) and 96 h (Fig. 7C and 7E). In addition, cell numbers were decreased with statistical significance in 0.32 μM CsA-treated P19 cells as differentiation proceeded, at 96 h (Fig. 7F).

These results demonstrate that CsA treatment during EB formation by P19 cells induces cell apoptosis and death.

## Discussion

### CsA induces cardiac differentiation but severely inhibits hemato-endothelial cell differentiation during EB formation

Addition of 0.8–2.5 μM (1–3 μg/mL) CsA to Flk1+ mesodermal cells potently increased the FCV cardiac progenitor population as well as cardiomyocyte differentiation in ES [14] and iPS cells [16]. However, CsA did not have any influence on Flk1+ mesoderm cell generation from undifferentiated ES [14] or iPS cells [16], indicating that the cardiomyocyte inducing activity of CsA is restricted to the period after mesoderm formation. In contrast, we found that addition of 0.32 μM (0.4 μg/mL) CsA to undifferentiated P19 cells during the EB formation stage more efficiently induces cardiac differentiation of P19 cells compared to adding CsA after EB formation. Our findings and previous reports suggest that the effect of CsA on cardiac differentiation may be affected by different factors such as cell type, cell source, differentiation stage of cells, and culture conditions.

In this study, mRNA expression levels of the endothelial lineage markers Flk1, Flt1, Flt4, Tie1, and Tie2, as well as the hematopoietic lineage markers Er71, Fli1, Gata1, Gata2, Sox17, Tal1, Lmo2, and Runx1 were severely reduced by CsA treatment during EB formation. Enforced expression of Er71 in ES cells resulted in a robust induction of Flk1+ mesoderm and enhanced hematopoietic and endothelial cell generation [36], whereas critical hemato-endothelial genes such as Tal1, Fli1, and Gata2 were severely downregulated in Er71-null Flk1+ cells [37], demonstrating that Er71 regulates Flk1+ mesoderm, blood, and vessel development. Furthermore, Rasmussen et al. [38] reported that Er71 is a critical regulator of mesodermal fate decisions that acts to specify the hematopoietic and endothelial lineages to the exclusion of cardiac lineages. In addition, Liu et al. [39] reported that Er71 specifies Flk1+ hemangiogenic mesoderm by inhibiting cardiac mesoderm and Wnt signaling, providing the molecular basis for the antagonistic relationship between hemangiogenic and cardiogenic mesoderm specification by Er71 and Wnt signaling. Our data and previous reports suggest that inhibition of the Er71- and Flk1-dependent signaling pathway by CsA during EB formation blocks hemato-endothelial differentiation and triggers activation of Wnt signaling genes, resulting in specific cardiac differentiation of P19 cells.

### CsA induces cardiac differentiation via upregulation of Wnts and cardiac mesodermal markers

Yan et al. [14] reported that FK506 and an NFAT inhibitor, 11R-VIVIT, showed no significant effect on cardiomyocyte induction of Flk1+ mesodermal cells, indicating that the main cardiogenic effect of CsA should be NFAT independent. However, we found that expression levels of Nfatc1 and Nfatc2 mRNA were significantly downregulated in 0.32 μM CsA-treated P19 cells (Fig. 6A), suggesting that the inhibition of NFATc transcription factors by CsA during EB formation could be involved in cell fate determination towards a cardiac cell lineage. Li et al. [30] reported that the calcineurin-NFAT signaling is both necessary and sufficient to switch ES cells from an undifferentiated state to lineage-specific cells, providing evidence for the regulatory role of the calcineurin-NFAT signaling in the balance between ES cell self-renewal and early lineage specification [30]. Interestingly, we found that CsA treatment significantly downregulated mRNA expression levels of Oct4 and Nanog, as well as the percentages of Oct4+ cells during EB formation, but their expressions increased, conversely, at 96 h in 0.32 μM CsA-treated P19 cells compared to control cells (Fig. 6). Our findings and the previous results suggest that CsA may finely adjust maintenance of stemness as well as induction of differentiation via inhibition of the calcineurin/NFAT signaling pathway in a dosage- and differentiation stage-dependent manner.

In this study, we demonstrated for the first time that CsA induces cardiac differentiation of P19 cells via activation of Wnts, such as Wnt3a, Wnt5a, and Wnt8a during EB formation. Wnt/β-catenin signaling is well known to be an essential regulator of cardiac differentiation and development [23,40–42]. Similarly, Huang et al. [43] reported that NFAT proteins repress Wnt signaling in HEK293T cells and also participate in regulating neural progenitor cell proliferation and differentiation in the neural tube of chick embryos. Our findings and the previous observation suggest that cross-talk between NFAT and Wnt signaling is involved in the proliferation and differentiation of stem cells.

In this study, we also found that Mesp1, Mesp2, and Mixl1 are significantly upregulated at 48 h by CsA treatment. In accordance with our result, Lindsley et al. [44] reported that Mesp1 generates mesoderm progenitors with cardiovascular, but not hematopoietic, potential in mouse ES cells independently of Wnt signaling. In addition, Bondue et al. [45] reported that Mesp1 acts as a key regulator at the top of the hierarchy of the gene network responsible for cardiovascular cell-fate determination. Our observation and previous reports suggest that CsA-induced Mesp transcription factors may activate another route to drive differentiation toward cardiac mesoderm, resulting in efficient cardiac mesoderm specification synergistically with a Wnt mediated-cardiac mesoderm specification pathway. Moreover, Mixl1 is also known as a key regulator in mesendoderm patterning during embryogenesis [46,47].

It is well known that fine crosstalk between the Wnt/catenin and BMP/Smad signaling pathways is important for mesoderm induction and subsequent cardiomyogenesis in stem cells [42,48]. Furthermore, Zhang et al. [49] reported that short-term BMP4 treatment initiates mesoderm induction in human ES cells via endogenous FGF and TGF-beta/Nodal/activin signaling activities. In this study, however, significant induction of TGF-β superfamily signaling pathway molecules such as BMP/Smad, Nodal and GDF1 were not found in CsA-treated P19 cells during EB formation. Furthermore, T gene, a key mesodermal factor, was not significantly induced by CsA alone. We found that CsA alone induces modest cardiac differentiation (~5-fold increase at the mRNA level) during EB formation compared to the untreated controls (Fig. 3B). However, combined treatment of CsA with DMSO significantly increased cardiac differentiation efficiency at the mRNA level (7.7-fold) and the protein level (4.2-fold) as indicated by levels of α-MHC, a cardiac marker, compared to CsA alone treatment, via upregulation of Wnt molecules and cardiac mesodermal markers (Fig. 3). Based on our findings and previous reports, we speculate that the relatively low efficiency of cardiac differentiation by CsA alone may be due to a lack of BMP/Smad signaling, and the subsequently incomplete coordination of Wnt/catenin and BMP/Smad signaling may also result in only partial activation of cardiac mesoderm markers.

### CsA modulates expression of p53 and Cyclins and induces cell death during EB formation

In this present study, we found that CsA significantly modulates the mRNA expression levels of Cyclins D1, D2, and E2, and p53 at 48 h post-EB formation (Fig. 7A). Furthermore, CsA induced cell death and reduced cell numbers during EB formation by P19 cells. In accordance with our observation, several in vitro and in vivo studies have reported that NFATc transcription factors regulate the cell cycle [50,51]. Similarly, Zhu et al. [52] reported that the expression of p53 mRNA and protein and apoptosis were significantly increased at 48 h during icariin-induced cardiac differentiation of mouse ES cells. Moreover, p53 induced differentiation of mouse ES cells by suppressing Nanog expression while inducing efficient p53-dependent cell-cycle arrest and apoptosis [53]. Hadjal et al. [54] also reported that p53 downregulation leads to a strong inhibition of the mesodermal master genes T and Mesp1, which affect cardiomyogenesis and skeletal myogenesis of ES cells. Our result and the previous studies demonstrate that p53 is an important regulator in stem cell differentiation.

Taken together, these results demonstrate that CsA induces the cardiac differentiation of P19 cells via activation of Wnt signaling pathway molecules. The cardiac mesoderm genes, Mixl1, Mesp1, and Mesp2 are key players for specification into cardiomyocyte differentiation, whereas Flk1 reduction is responsible for inhibition of hemato-endothelial differentiation, possibly via Er71-mediated signaling pathways in CsA-induced P19 cells. An efficient cardiac differentiation protocol achieved by the combined administration of CsA and DMSO would contribute to elucidating the molecular mechanisms underlying the differentiation of stem cells to cardiac lineages.

## Supporting Information

S1 Fig Effects of different concentrations of CsA or 1% DMSO treatment on EB formation by P19 cells. (A) Effects of different concentrations of CsA during EB formation by P19 cells. (B) Effects of different concentrations of CsA plus 1% DMSO during EB formation by P19 cells. (C) EBs formed by 10 μM CsA-treated P19 cells show adhesive (arrowheads) and distorted (arrows) morphology. Scale bars = 200 μm.
(TIF)

S2 Fig TGF-β superfamily signaling pathway molecules were not significantly affected by CsA treatment during EB formation by P19 cells. (A) Real-time PCR result showing no modulation of BMP/Smad signaling pathway molecules in P19 cells treated with 0.32 μM CsA during EB formation. (B) Real-time PCR result showing no modulation of Gdf1 or Nodal signaling pathway molecules in P19 cells treated with 0.32 μM CsA during EB formation.
(TIF)

S3 Fig mRNA expression levels of endodermal lineage markers were not significantly affected by 0.32 μM CsA treatment during EB formation by P19 cells. (TIF)

S4 Fig Morphology of EBs treated with 0.32 μM CsA, 1% DMSO, or CsA plus DMSO for 48 (A) or 96 h (B). Scale bars = 200 μm.
(TIF)

S5 Fig Flk1 expression is reduced in CsA treated-EBs. Immunofluorescence staining showing reduced expression of Flk1 in dissociated cells from EBs treated with 0.32 μM CsA for 48 (A) and for 96 h (B). Scale bars = 20 μm.
(TIF)

S1 Table Primers used for real-time PCR. (DOC)

S1 Video Beating cell clusters generated in P19 cells treated with 0.32 μM CsA at day 16. (MP4)

S2 Video Beating cell clusters generated in P19 cells treated with 0.32 μM CsA plus 1% DMSO at day 16. (MP4)

S3 Video Beating cell clusters generated in P19 cells treated with 1% DMSO at day 16. (MP4)
