# Supplementary figures and images for: Cyclosporin A Induces Cardiac Differentiation but Inhibits Hemato-Endothelial Differentiation of P19 Cells
Source: PLoS One. 2015 Jan 28;10(1):e0117410. doi: 10.1371/journal.pone.0117410 (PMC4309530; doi:10.1371/journal.pone.0117410)

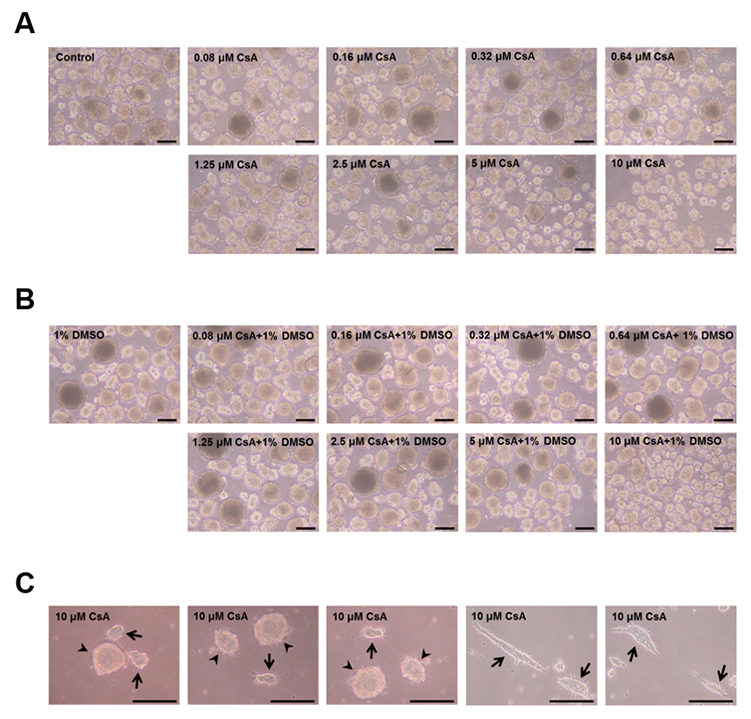

Supplement: S1 Fig — (A) Effects of different concentrations of CsA during EB formation by P19 cells. (B) Effects of different concentrations of CsA plus 1% DMSO during EB formation by P19 cells. (C) EBs formed by 10 μM CsA-treated P19 cells show adhesive (arrowheads) and distorted (arrows) morphology. Scale bars = 200 μm. (TIF) [file pone.0117410.s001.tif]

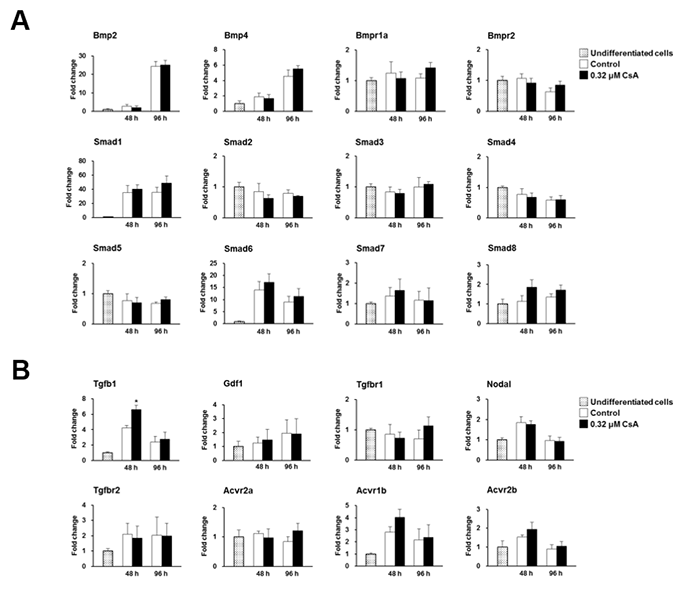

Supplement: S2 Fig — (A) Real-time PCR result showing no modulation of BMP/Smad signaling pathway molecules in P19 cells treated with 0.32 μM CsA during EB formation. (B) Real-time PCR result showing no modulation of Gdf1 or Nodal signaling pathway molecules in P19 cells treated with 0.32 μM CsA during EB formation. (TIF) [file pone.0117410.s002.tif]

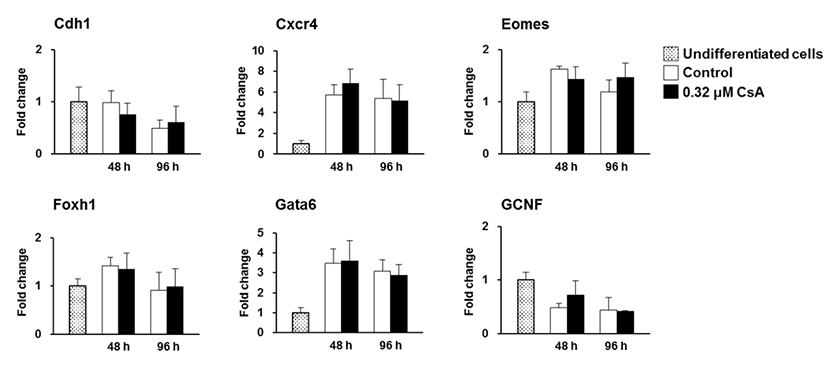

Supplement: S3 Fig — (TIF) [file pone.0117410.s003.tif]

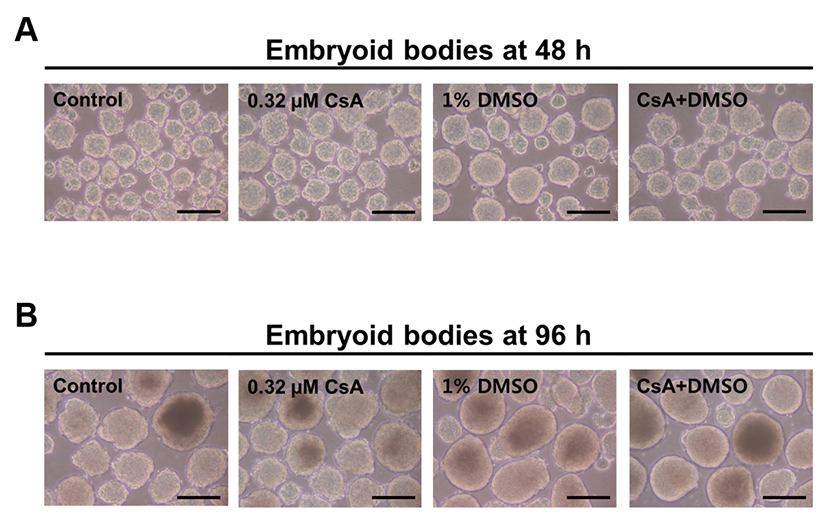

Supplement: S4 Fig — Scale bars = 200 μm. (TIF) [file pone.0117410.s004.tif]

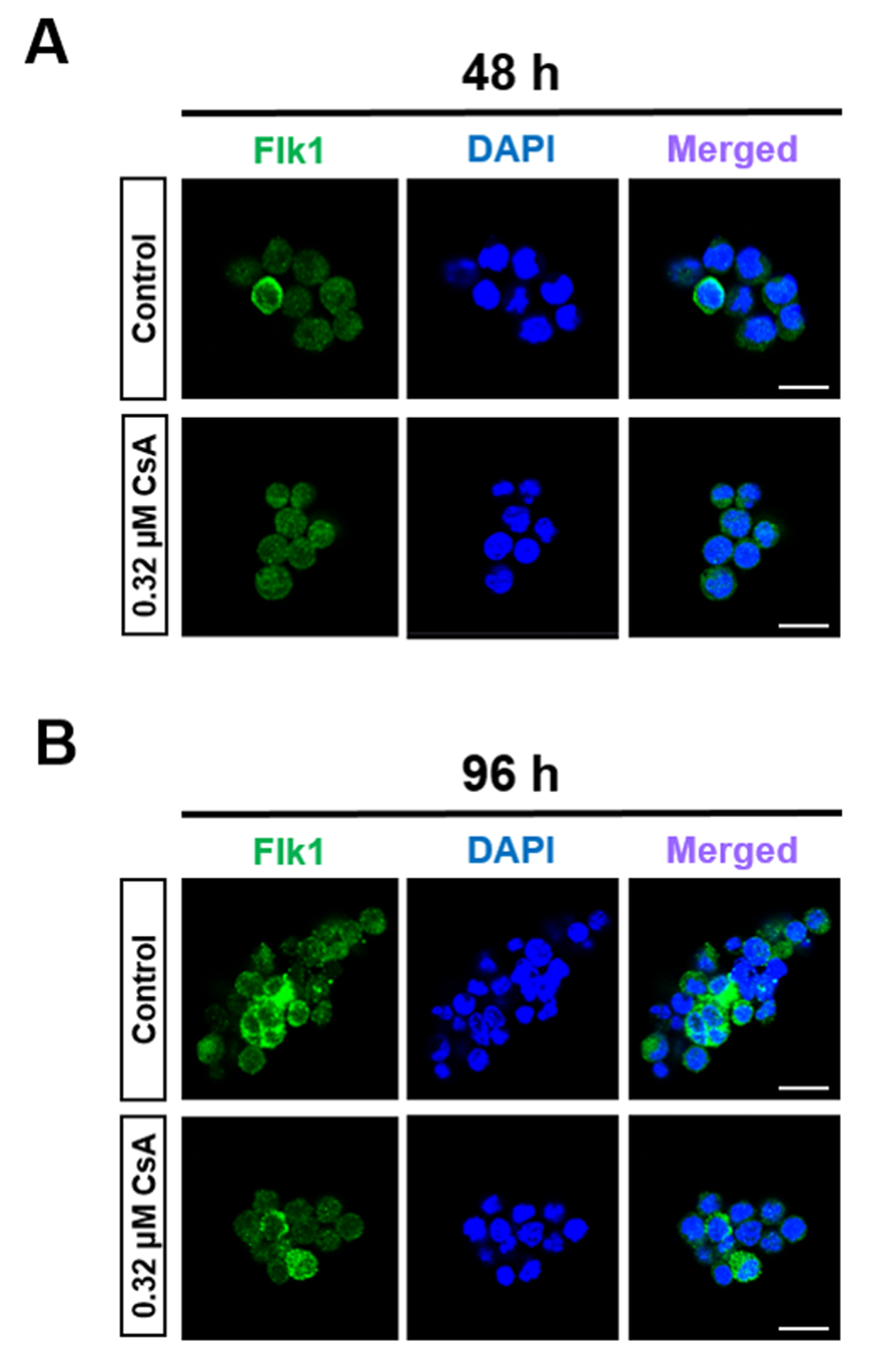

Supplement: S5 Fig — Immunofluorescence staining showing reduced expression of Flk1 in dissociated cells from EBs treated with 0.32 μM CsA for 48 (A) and for 96 h (B). Scale bars = 20 μm. (TIF) [file pone.0117410.s005.tif]
