# Supplementary material for: Cyclosporin A Induces Cardiac Differentiation but Inhibits Hemato-Endothelial Differentiation of P19 Cells
Source: PLoS One. 2015 Jan 28;10(1):e0117410. doi: 10.1371/journal.pone.0117410 (PMC4309530; doi:10.1371/journal.pone.0117410)
Supplement: S1 Table — (DOC) [file pone.0117410.s006.doc]

| | **Table S1** Primers used for real-time PCR. | | |  | | --- | --- | --- | --- | | **Gene** | **Forward primer (5'-3')** | **Reverse primer (5'-3')** | **Product (bp)** | | **α-MHC** | GGATTCTCTGAAAAGTTAAC | GGCGTTCCTTCTCTGACTTT | 108 | | **Acvr2a** | GCTTCTCGTTGCACTGCTG | TGCACAACAACTTCCTGCATA | 110 | | **Acvr1b** | CCTCCTTCTTCCCCCTTGT | TTGGTCTGTAGGCAGCTGGT | 106 | | **Acvr2b** | GGAAGCCTCCTGGGGATAC | CCATGGCGTACATGTCGAT | 123 | | **AP** | CTGACTGACCCTTCGCTCTC | GTGGTCAATCCTGCCTCCT | 105 | | **Bmp2** | CGGACTGCGGTCTCCTAA | CCCAGCTCTGGAATGAGG | 113 | | **Bmp4** | GAGGAGTTTCCATCACGAAGA | GCTCTGCCGAGGAGATCA | 122 | | **Bmpr1a** | TTCATCATTTCTCATGTTCAAGG | TCTGGTGCTAAAGTCACTCCATT | 113 | | **Bmpr2** | TTGGATACAGAATGTTGACAGGA | TTCAGGTTATCCAGGTCAAGG | 103 | | **CD31** | AGAGACGGTCTTGTCGCAGT | TACTGGGCTTCGAGAGCATT | 152 | | **Cdh1** | GCTCTCATCATCGCCACAG | GATGGGAGCGTTGTCATTG | 96 | | **Cdk1** | CTCATCTTTGAGTTCCTGTCCA | CCCTGGAGGATTTGGTGTAA | 113 | | **Cdk2** | CCCTGTCCGAACTTACACTCA | CCCAGGCTCCAGATATCCA | 108 | | **Cdk4** | GCCAGAGATGGAGGAGTCTG | CCTTGTGCAGGTAGGAGTGC | 110 | | **Cdk6** | GGATCAACTAGGAAAAATCTTGGA | ATGGGTTGAGCAGATTTGGA | 111 | | **cTnT** | GGCTCACTTCGAGAACAGGA | TCATTGCGAATACGCTGCT | 108 | | **Cxcr4** | TGGAACCGATCAGTGTGAGT | GGGCAGGAAGATCCTATTGA | 131 | | **Cyclin D1** | CC TTTGTGGCCC TCTGTG | CAGG TTCAGGCCTT GCAT | 101 | | **Cyclin D2** | ATCGCTCTGTGCGCTACC | GTGTTCACTTCATCATCCTGCT | 113 | | **Cyclin D3** | CGTGCAAAAGGAGATCAAGC | AGCCAGAGGGAAGACATCCT | 103 | | **Cyclin E1** | CTGAGAGATGAGCACTTTCTGC | GAGCTTATAGACTTCGCACACCT | 102 | | **Cyclin E2** | GCCATCGACTCTTTAGAATTTCA | TGTCATCCCATTCCAAACCT | 109 | | **Eomes** | CAAAGCGGACAATAACATGC | TGGTGAGTTTTAACTTCCCAAAG | 113 | | **Er71** | CTCGCTACTCCAAAACTAACCAC | TGGAACTCGCGGCTATTG | 121 | | **Fli1** | AGACCATGGGCAAGAACACT | GCCCCAGGATCTGATAAGG | 60 | | **Flk1** | GGCGGTGGTGACAGTATCTT | GTCACTGACAGAGGCGATGA | 162 | | **Flt1** | GCAAACGTGACTTATTCTGTCTCA | CTGTCTAGGCGGGGCTTC | 109 | | **Flt4** | GAGATCCTGGGGGACCTG | CCTGCATGAAGCCATCCT | 106 | | **Foxf1a** | TTCACCAAAACAGTCACAACG | CCATGGCATTGAAAGAGAAGAC | 114 | | **Foxh1** | CTTCCGCAGGCTGAAACT | GAAAGGTTGTGGCGGATG | 108 | | **GAPDH** | ACCACCATGGAGAAGGC | GGCATGGACTGTGGTCATGA | 236 | | **Gata1** | GCATCAACAAGCCCAGGT | AAACTGGGGCAAGGGTTCT | 89 | | **Gata2** | GCTTCACCCCTAAGCAGAGA | TGGCACCACAGTTGACACA | 72 | | **Gata6** | AAAGCTTGCTCCGGTAACAG | ATCACTGATGCCCCTACCC | 134 | | **GCNF** | TTGGGCACACCTATGTTGATT | CCAGGCAATCTGCCTAAAGA | 108 | | **Gdf1** | CACTCTGGAAGCTCAGACAGC | AGGTAGGATGGGATAGAGTCCTG | 131 | | **GFAP** | GGATTTGGAGAGAAAGGTTGAA | AGCTGCTCCCGGAGTTCT | 93 | | **Lmo2** | CGAAAGGAAGAGCCTGGAC | AGCGGTCCCCTATGTTCTG | 107 | | **LPL** | TCTGGGCTATGAGATCAACAAG | TCTTGACTTGGTAATGGAACACTT | 104 | | **MBP** | AGCCCTCTGCCCTCTCAT | GGTAGTTCTCGTGTGTGAGTCCT | 75 | | **Mesdc2** | TCACCGTGTCTGGGAACC | TCGGATCCCACGATGAAC | 112 | | **Mesp1** | ACCCATCGTTCCAGTACGC | AGCATGTCGCTGCTGAAGA | 91 | | **Mesp2** | CCCAGAGCCTAGGAACAAGA | TCTGGAGACACAGAAAGACTCTGA | 93 | | **Mixl1** | CCATGTACCCAGACATCCACT | CGGTTCTGGAACCACACCT | 88 | | **Nanog** | CACCCACCCATGCTAGTCTT | ACCCTCAAACTCCTGGTCCT | 150 | | **Nfatc1** | TCTCACCACAGGGCTCACTA | CATTCTCCAAGTAACCGTGTAGC | 103 | | **Nfatc2** | CCAGACCTACCTGGATGACG | TAGAAGGCGTCGCGAGATTA | 105 | | **Nfatc3** | CCGACTTGTATTTCGTGTGC | TGAGCAGATCGCTGAGAGC | 99 | | **Nfatc4** | CGGCATGGATTACCTAGCAG | GGGAGGTAGGGCAGAGGT | 97 | | **NF-H** | TGAAAAGCACCAAGGAGTCA | GCTGCTGAATAGCGTCCTG | 96 | | **Nodal** | TGTGAGGGCGAGTGTCCTA | TGGGGTTGGTATCGTTTCAG | 92 | | **Oct4** | GGCGTTCTCTTTGGAAAGGTGTTC | CTCGAACCACATCCTTCTCT | 312 | | **Osteopontin** | CCCGGTGAAAGTGACTGATT | TTCTTCAGAGGACACAGCATTC | 142 | | **p21** | CAGATCCACAGCGATATCCA | GGCACACTTTGCTCCTGTG | 94 | | **p27** | GAGCAGTGTCCAGGGATGAG | TCTGTTCTGTTGGCCCTTTT | 77 | | **p53** | GATGTTCCGGGAGCTGAAT | AGACTGGCCCTTCTTGGTCT | 112 | | **Pdgfr-α** | GGATGATCTGCAAGCATATTAAGAA | AGGACACTCGTCCCTCCAC | 129 | | **PPARγ2** | TGCTGTTATGGGTGAAACTCTG | CTGTGTCAACCATGGTAATTTCTT | 110 | | **Runx1** | CTCCGTGCTACCCACTCACT | ATGACGGTGACCAGAGTGC | 102 | | **SM α-actin** | CAACCGGGAGAAAATGACC | CAGTTGTACGTCCAGAGGCATA | 107 | | **Smad1** | AGCCCAACAGCCACCCGT | GCAACTGCCTGAACATCTCC | 259 | | **Smad2** | AGGACGGTTAGATGAGCTTGAG | GTCCCCAAATTTCAGAGCAA | 95 | | **Smad3** | TCAAGAAGACGGGGCAGTT | CCGACCATCCAGTGACCT | 98 | | **Smad4** | ATTGGATGGACGACTTCAGG | TGCTTTAGTTCATTCTTGTGTAGATCA | 102 | | **Smad5** | CAACTCCCCAGCAAGCTC | GGAATTATCTGGGGCCATCT | 106 | | **Smad6** | TCCTGACCAGTACAAGCCACT | TTCACCCGGAGCAGTGAT | 94 | | **Smad7** | GGGGGAACGAATTATCTGG | ACCACGCACCAGTGTGAC | 86 | | **Smad8** | TCCAGCAGTCTCTCTGTCCG | GTGCTGGGGTTCCTCGTAG | 164 | | **Smoothelin** | CCGACCAAACTAACACGAAAC | ACTCAGAATTCCTGACATGTGG | 111 | | **Sox17** | GGTCTGAAGTGCGGTTGG | TGTCTTCCCTGTCTTGGTTGA | 109 | | **T** | AGTGGGCCTGGAGGAGAG | TTCAGCACCGGGAACATC | 99 | | **Tal1** | GCTCGCCTCACTAGGCAGT | CTCTTCACCCGGTTGTTGTT | 78 | | **Tgfb1** | CCTTCCTGCTCCTCATGG | CGCACACAGCAGTTCTTCTC | 121 | | **Tgfbr1** | AGAAGAGCGTTCATGGTTCC | CGTCCATGTCCCATTGTCT | 112 | | **Tgfbr2** | CAGCTTCTGGCTCAACCAC | CGGATGCTCCAGCTCACT | 130 | | **Tie1** | CAGGCACAGCAGGTTGTAGA | GTGCCACCATTTTGACACTG | 160 | | **Tie2** | AAGCATGCCCATCTGGTTAC | GCCTGCCTTCTTTCTCACAC | 138 | | **VE-cadherin** | CTCCCCTCCTGAGGCAAT | CGTGGAGGAGCTGATCTTG | 103 | | **Wnt2a** | GTCCCTCTCGGTGGAATCT | AGCCACCTGTAGCTCTCATGTA | 100 | | **Wnt3a** | GGAGTGCCAGCACCAGTT | GCATGGACAAAGGCTGACTC | 117 | | **Wnt5a** | TGCCACTTGTATCAGGACCA | GTCTCTCGGCTGCCTATTTG | 158 | | **Wnt7a** | CGAGAGCTAGGCTACGTGCT | CAGCACATGAGGTCACAGCC | 263 | | **Wnt7b** | TCATGAACCTTCACAACAATGA | TGGTCCAGCAAGTTTTGGT | 114 | | **Wnt8a** | CAGTTTTCAACCCACAACAGG | CTGCAGTTCTTGGTGACTGC | 104 | | **Wnt9a** | ACCTCGTGGGTGTGAAGGT | ACCTCGTGGAAGGGTGCTA | 118 | | **Wnt11** | ACCTGCTTGACCTGGAGAGA | TTATTGGCTTGGGATCCTGT | 262 | |  |
| --- | --- | --- | --- | --- | --- | --- | --- | --- | --- | --- | --- | --- | --- | --- | --- | --- | --- | --- | --- | --- | --- | --- | --- | --- | --- | --- | --- | --- | --- | --- | --- | --- | --- | --- | --- | --- | --- | --- | --- | --- | --- | --- | --- | --- | --- | --- | --- | --- | --- | --- | --- | --- | --- | --- | --- | --- | --- | --- | --- | --- | --- | --- | --- | --- | --- | --- | --- | --- | --- | --- | --- | --- | --- | --- | --- | --- | --- | --- | --- | --- | --- | --- | --- | --- | --- | --- | --- | --- | --- | --- | --- | --- | --- | --- | --- | --- | --- | --- | --- | --- | --- | --- | --- | --- | --- | --- | --- | --- | --- | --- | --- | --- | --- | --- | --- | --- | --- | --- | --- | --- | --- | --- | --- | --- | --- | --- | --- | --- | --- | --- | --- | --- | --- | --- | --- | --- | --- | --- | --- | --- | --- | --- | --- | --- | --- | --- | --- | --- | --- | --- | --- | --- | --- | --- | --- | --- | --- | --- | --- | --- | --- | --- | --- | --- | --- | --- | --- | --- | --- | --- | --- | --- | --- | --- | --- | --- | --- | --- | --- | --- | --- | --- | --- | --- | --- | --- | --- | --- | --- | --- | --- | --- | --- | --- | --- | --- | --- | --- | --- | --- | --- | --- | --- | --- | --- | --- | --- | --- | --- | --- | --- | --- | --- | --- | --- | --- | --- | --- | --- | --- | --- | --- | --- | --- | --- | --- | --- | --- | --- | --- | --- | --- | --- | --- | --- | --- | --- | --- | --- | --- | --- | --- | --- | --- | --- | --- | --- | --- | --- | --- | --- | --- | --- | --- | --- | --- | --- | --- | --- | --- | --- | --- | --- | --- | --- | --- | --- | --- | --- | --- | --- | --- | --- | --- | --- | --- | --- | --- | --- | --- | --- | --- | --- | --- | --- | --- | --- | --- | --- | --- | --- | --- | --- | --- | --- | --- | --- | --- | --- | --- | --- | --- | --- | --- | --- | --- | --- | --- | --- | --- | --- | --- | --- | --- | --- | --- | --- | --- | --- | --- | --- | --- | --- | --- | --- | --- | --- | --- | --- | --- | --- | --- | --- | --- | --- | --- | --- | --- | --- | --- | --- | --- | --- | --- | --- | --- | --- | --- | --- | --- | --- | --- | --- |
